# Lock-Out Valve to Decrease Catheter-Associated Urinary Tract Infections

**DOI:** 10.1155/2014/765756

**Published:** 2014-01-20

**Authors:** Amir Shbeeb, Jennifer L. Young, Scott A. Hart, Juliet C. Hart, Joel Gelman

**Affiliations:** ^1^Department of Anesthesiology, University of Southern California Medical Center, 1200 N. State Street, IPT, Room C4E100, Los Angeles, CA 90033, USA; ^2^The Urology Group, 19415 Deerfield Avenue, Suite 112, Leesburg, VA 20176, USA; ^3^San Diego, CA 92121, USA; ^4^Center for Reconstructive Urology, 333 City Boulevard West, Suite 1240, Orange, CA 92868, USA

## Abstract

Patients with long-term indwelling urinary catheters are at an increased risk for urinary tract infection due to bacteriuria. Catheter-associated urinary tract infections (CAUTIs) are a significant source of morbidity and mortality in long-term care facilities as well as in ambulatory patients requiring long-term catheterization. There is increased interest in the financial impact of CAUTI as Medicare no longer provides reimbursement for nosocomial CAUTIs. Ascending bacteria may in part enter the closed drainage system when the patient switches between leg and night collection bags. In an attempt to reduce this ascent, a double valve lock-out system was devised that maintains a closed system during bag exchange. The concept is introduced and CAUTIs are reviewed.

## 1. Introduction

The urinary catheter is a device that serves as a tube to mechanically drain the bladder for a variety of pathological conditions or surgical procedures. Methods of collection for urinary catheterization include intermittent catheterization, condom or Texas catheters, adherent urine collection bags, and indwelling urethral or suprapubic catheters.

Each type of urinary catheters has its own indications and associated risks and benefits. Indwelling urinary catheters have the highest risk of nosocomial infection due to the fact that they remain in the bladder for a long period of time and allow microbial colonization and invasion [1]. In general, catheter-associated urinary tract infection (CAUTI) is the most common nosocomial infection in the United States, accounting for nearly a third of all hospital infections [2, 3]. In fiscal year 2006, there were 11,780 Medicare cases of CAUTI with an average Medicare payment for admission in which CAUTI was present of $40,347 [4]. However, as of 2008, Medicare will no longer be reimbursing for CAUTI [5]. Reduction of CAUTI would decrease morbidity, mortality, length of hospital stay, and overall healthcare cost [6].

There is a daily infection rate of 5% in patients with long-term (>30 days) indwelling catheters [1]. One study showed that, after 8 weeks, 113 out of 115 patients with urinary catheters were infected [1]. Of note, the remaining 2 patients that were not infected were on antibiotics at that time. Urinary tract infection occurs when bacteria bypass normal host defenses [7] and gain access to the bladder while avoiding the urothelium's bactericidal peptides, cytokines, defensins, and adhesion molecules of the urothelium [8]. Bacteria gain access to the urinary tract via two routes: from within the catheter or from the outside of the catheter on the periurethral mucosal surface. Ambulatory patients that consistently change their urinary bag from a leg bag to a large night bag are more prone to infections resulting from bacteria ascending within the catheter [9].

A lower incidence of CAUTIs is not only beneficial for the individual patient but also decreases the number of patients requiring antimicrobial treatment and subsequent microbial drug resistance. Maintenance of a closed urinary drainage system has been shown to be one of the most successful ways to prevent urinary tract infections [9–12].

Currently, ambulatory patients that regularly change urine collection bags temporarily violate the closed drainage system during bag exchange from daytime leg bags to large nighttime bags. These patients would benefit from a system to ensure a closed drainage system including during bag exchange. Wenzler-Röttele et al. inoculated two sets of drainage bags with *Escherichia coli*; one bag had a single valve between drainage tube and the catheter, and the other set had a double nonreturn valve model system. The time was measured from the point of inoculation to the point of colonization of the drainage port, which is just proximal to the bladder model, and compared between the two sets of drainage bags. They found that it took 14 days to colonize the single valve system while it took more than 21 days to colonize the double nonreturn valve system. They concluded that the double nonreturn valve increased the time necessary for bacterial colonization of the catheter, suggesting that a double nonreturn valve system is able to be used on a long term without increasing the risk of infection in a catheterized bladder model with twice daily bag emptying [13]. This paper introduces an approach to improve the integrity of the closed urinary drainage system.

## 2. Materials and Methods

Patient S.H. is a Principal Scientist at a biotechnology therapeutics company. He required management with a suprapubic catheter for several months due to urethral stricture disease and found switching between his leg and night bags worrisome for violation of the sterile collecting system. He was also troubled by the spray of urine that resulted from the tubing popping off the bags and therefore devised an autoclavable device to overcome these concerns.

**Figure 1:**
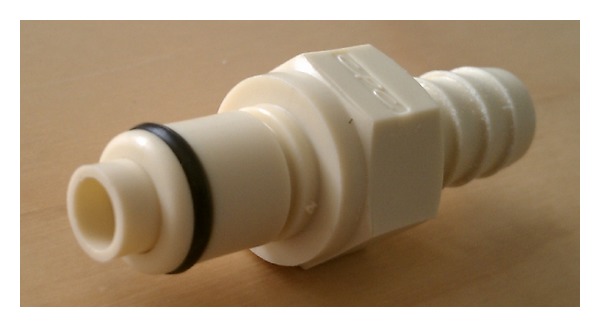
Polypropylene quick-disconnect tube coupling plug. Compatible with 3/8 inch internal diameter medical-grade tubing.

**Figure 2:**
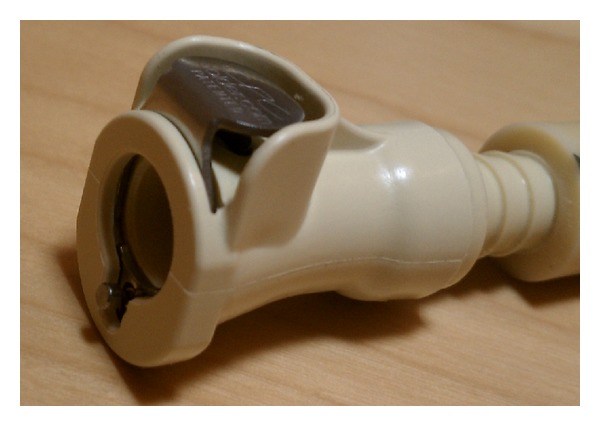
Polypropylene quick-disconnect tube socket with valve end-on.

**Figure 3:**
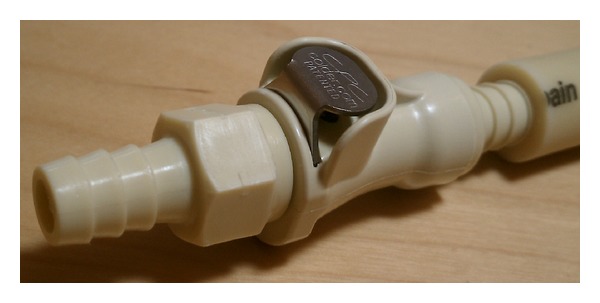
Polypropylene quick-disconnect tube coupling plug and tube socket with double lock-out valve, manufactured by Colder Products Company.

**Figure 4:**
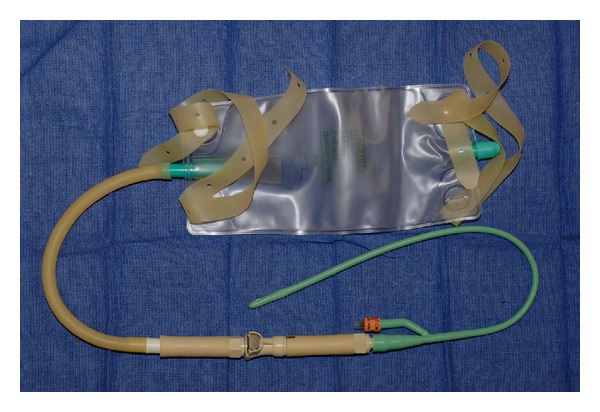
Lock-out valve connected with catheter and leg drainage bag.

He first obtained a quick-disconnect valve which was designed to shut off flow of liquid from both ends while disconnected. In the patient's lab, the valve was used to prevent leakage when liquids needed to flow through a conduit intermittently. The specific polypropylene plug (Figure 1) and socket (Figure 2) components used were autoclavable and readily available from hardware supplier, McMaster-Carr (number 51545K91 [plug] and number 51545K74 [socket]). Colder Products Company is the manufacturer of the parts (number PLCD1700612 [plug] and number PLCD2200612 [socket]) (Figure 3). Both sides of the quick-disconnect valve were then configured with plastic connectors (Figure 4) so that they are compatible with medical grade tubing with a 3/8 inch internal diameter. The plastic connector from the valve was then attached to the suprapubic drain tube. Thick-walled silicone tubing, which would not be distorted during body movements, was then used to link the plastic connector on the valve to the drainage bag. The body of the socket contains a metal spring-loaded button that, when pressed, releases the valve connection and blocks the escape of fluid through the valve. At this point, the valve is separated and the urinary bag can be emptied, while maintaining a closed drainage system.

To reattach the drainage bag to the drain tube, the valve between the catheter and the drainage bag is simply reconnected, and a click is heard. When both a leg bag and a night bag are outfitted with a valve plug, either bag type can readily be connected to the valve socket on his drainage catheter.

## 3. Comment

This lock-out valve design may provide a number of advances over the current indwelling catheter system in use in the United States. Maintenance of a closed drainage system while changing from a large night bag to a leg bag may decrease the introduction of bacteria into the system. Less chance of introduction of bacteria into the system could translate into a decreased incidence of CAUTI. Additionally, the lock-out valve may reduce spillage of the bag contents, creating a cleaner environment for the patient, caregivers, and surroundings. It may be difficult to quantify the health outcomes from this but certainly decreased spillage of urine is desirable.

On review of the urology literature and current patents, one similar device was identified. Patent 5,496,300 describes the mechanics and engineering of a coupling device in order to prevent fluid spillage and to avoid contaminants from entering the tube during bag exchange. This is a device that couples a urinal to a urine collection bag. A quick release coupling readily connects and disconnects the flexible tube to the urine collection bag to provide a fluid passageway of urine from the collection bag into the urinal upon connection. The coupling closes both the tube opening and the bag opening upon disconnection to prevent fluid from spilling out and to avoid contaminants from entering the flexible tube. However, this patent is currently expired according to United States Patent and Trademark Office OG Notices: May 4, 2004. To our knowledge, a coupling device of this nature is not available.

Since the entrance of bacteria into the bladder can be intraluminally and extraluminally (between catheter and urethral mucosa), this device can only prolong the time for the intraluminal access of the bacteria into the bladder. Only a clinical study could demonstrate the benefit of such a device. We feel this would be justified to evaluate a potential great technical improvement to the indwelling catheter.

## 4. Conclusions

This valve system is easy to use with minimal technical training required. It would be feasible to use in all patients with long-term catheters that switch between leg and night bags to preserve the closed drainage system and possibly reduce CAUTIs. The disadvantage would be the increased cost of the valve. Further investigation is needed to determine the exact benefits and cost effectiveness of this device.
